# Biodegradation of Methanol Using Thiosulphate as an Electron Acceptor Under Anaerobic Conditions

**DOI:** 10.5696/2156-9614-6.12.61

**Published:** 2016-12-22

**Authors:** Mekonnen Maschal Tarekegn

**Affiliations:** Department of Environment and Climate Change Management, Ethiopian Civil Service University, Addis Ababa, Ethiopia

**Keywords:** methanol biodegradation rate, thiosulphate biodegradation rate, sulfur reducing bacteria, biomass growth rate

## Abstract

**Background.:**

Methanol is a volatile organic compound commonly found in the effluent of the pulp and paper industries. Because of its toxicity, methanol can cause metabolic acidosis, neurologic sequelae, and even death when ingested. Information on biokinetic activity such as biodegradation rate of methanol and thiosulphate, biomass growth rate and biomass yield coefficient is limited in the literature.

**Objectives.:**

To study the biomass growth rate and biomass yield coefficients of methanol and thiosulphate biodegradation. This research aims to increase knowledge of how to reduce the emission of toxic gas to the environment.

**Methods.:**

The biodegradation trends of both methanol and thiosulphate were studied under anaerobic conditions using batch experiments at ambient temperature and alkaline conditions. Both supplement each other for their degradation. Methanol is an electron donor, whereas thiosulphate acts as an electron acceptor. A mixed culture from a previously used biomass in a biotrickling filter reactor from theUnited Nations Educational, Scientific and Cultural Organization (UNESCO), International Graduate Water Education Facility and fresh activated sludge from the Harnaschpolder wastewater treatment plant were used as a biomass source.

**Results.:**

A specific biomass growth rate of biomass ranging from 0.04 to 1.7g per day was observed. The thiosulphate is biologically degraded by the biomass grown inside the reactor. The biodegradation rate of thiosulphate in the reactor varied from 0.02 to 0.80g per unit gram of biomass per day. A biodegradation rate of methanol in the reactor was observed in the range between 0.04 to 3.9g per unit gram of biomass per day. Bacterial biomass was grown as per the amount of methanol present inside the reactor. A maximum biomass yield coefficient of 0.7g biomass per gram of methanol was recorded. Thiosulphate was converted to sulphate that indirectly served as an electron acceptor for methanol degradation. Both degradation of methanol and thiosulphate in this experiment were in the range of the degradation rate shown for sulphate and organic compounds in other studies.

**Conclusion.:**

Simultaneous removal of thiosulphate and methanol using an anaerobic bioreactor is promising and can be applied on an industrial scale. This finding is an important contribution to public health as it reduces the emission of toxic gas to the environment.

## Introduction

Methanol gas is one of the main volatile organic gasses emitted from the pulp and paper industries, as well as the paint and petrochemical industries. About 80% of the chemical oxygen demand of pulp and paper industry wastewater is composed of methanol. Methanol is a highly volatile organic compound. Its concentration in the condensate of pulp and paper industry wastewater can reach 46 mg/L.^1^ With an increase in pulp and paper manufacturing, the emission of methanol gas can cause a serious greenhouse effect. Methanol can also be converted into formic acid when ingested into the human body and can cause blindness.^2^ It can also be converted to formate and further poison the optic disc, causing edema as well as intracellular problems and intra-axonal swelling. Formate transformed from methanol is responsible for metabolic acidosis, which indirectly causes serious ocular effects. Previous research has shown that abdominal, leg and back pain have also been observed as a delayed effect of methanol exposure. Irreversible damage from the effects of methanol has been found even 6 years after toxic exposure.^3^ According to study follow-up, 72% of sequelae involved new neurological and visual complications. According to a US Environmental Protection Agency report in 2015, methanol poisoning is dependent on exposure time. According to the report, the acute exposure limit of 670 ppm/V for 10 minutes and 270 ppm/V for 8 hours results in discomfort, 11,000 ppm/V for 10 minutes and 520 ppm/V for 8 hours causes irreversible or other serious, long-lasting effects, and an exposure limit of 1600 ppm/V leads to life-threatening effects or death.^4^

Methanol also causes serious aquatic ecological damage. In anaerobic environments, methanol can be used up by methanogenic, acetogenic, sulphate reducing or nitrogen reducing bacteria.^5^ Methanol can also be degraded by syntrophic cultures of anaerobic microorganisms.^6^ Acetate and butyrate are produced from methanol degradation in the presence of carbon dioxide (CO_2_) using homoacetogens.^1^,^7^ Methanogenicarchaea degrade methanol gas to methane and carbon dioxide.^8^ In the sulphidogenic process, methanol acts as an electron donor for sulphate reducing bacteria and is converted to CO_2_ while producing hydrogen sulphide either from sulphate or thiosulphate.^9^

**Abbreviations**
*CO_2_*: Carbon dioxide
*NPOC*: Non-purgeable organic carbon

Information on biokinetic activity such as biodegradation rate of methanol and thiosulphate, biomass growth rate and biomass yield coefficient is limited in the literature. In this study, batch experiments using mixed cultures were performed to study the biomass growth rate and biomass yield coefficients of methanol and thiosulphate biodegradation.

## Methods

This experiment was performed in duplicate using sealed laboratory grade 500 ml glass bottles. Biomass was detached from sponges of a biotrickling filter packing bed and fresh activated sludge was taken from the Harnaschpolder wastewater treatment plant in Holland. A biomass quantity of 12.5 ml (5% the volume of growing mineral solution) and 250 ml of growth mineral medium was mixed in a 500 ml bottle. Nitrogen gas was enriched to ensure an anaerobic environment in the bottle. The pH was adjusted to 8.0 with 5 molarsodium hydroxide and 5 molar hydrochloric acid solution followed by addition of 150 μl of 24.56 M methanol. Finally, the bottles were kept at a temperature of 20°C and agitated on a horizontal orbital shaker at 176 rpm. The gas and liquid samples were collected simultaneously for 10 days. A modified DSM 63 (Deutsche Sammlung von Mikroorganismen und Zellkulturen bacterial nutrient recipe) growth mineral medium composition was used.

The concentration of sulphate and thiosulphate was determined by ion chromatography methods^8^. The sample taken from the sampling port was filtered, diluted by ten and measured with an ion chromatography analytical instrument. Hydrogen sulphide gas in the reactor was regularly measured by gas chromatography. The concentration of sulphide ions in the liquid was determined by measuring the spectrophotometric absorbance on an ultraviolet spectrophotometer.

The quantity of methanol in the liquid was determined by quantifying the amount of non-purgeable organic carbon (NPOC) using a TOC-L Shimadzu total organic carbon analyser. The concentration of carbon dioxide was determined by gas chromatographic method. A standards of known carbon dioxide concentration ‘5% of CO_2_ greenhouse gas GC standard’ that was prepared by certified GC and calibration standard suppliers to UNESCO-IHE institute for water education were used for quantifying the amount of CO_2_ in the head space of the bottle. The peak area of known carbon dioxide gas concentration in the standard and the sample taken from the reactors head space were taken from the GC corresponding reading. 9,10 Finally, the concentration of CO_2_ was determined using Equations 1 and 2.

The Lowry protein assay explained in Kamlage B. 1996 was adapted to determine the biomass amount of liquid in the reactor. The techniques and procedures for protein assay using a spectrophotometer are described in the literature.^12^,^13^ First, the proteins of a sample biomass and bovine serum albumin standard solution were treated using an alkali solution of copper ion. A Folin reagent was added. The aromatic amino acids in the sample reduced the phosphomolybdatephosphotungstic acid present in the Folin reagent which forms a blue colour solution.


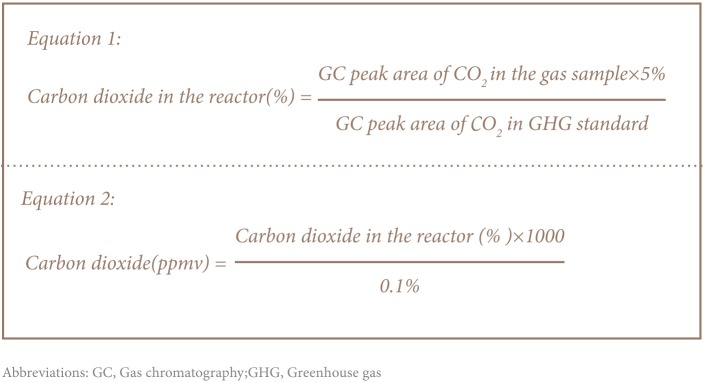


Depending on the intensity of colour, the amount of protein in the sample was estimated by reading the absorbance (at 750 nm) after 30 minutes of Folin reaction.

As shown in Equations 3 and 4, the degradation rate of both methanol and thiosulphate were determined by taking the change in concentration of substrate with change in concentration of biomass with time.

All quantitative continuous data collected from the batch experiment was analysed using Microsoft Excel (Redmond, WA, USA). Data collected from the reactors were organised and summarised in two-way tables on Microsoft Excel sheets. Means, standard deviations and standard errors were also calculated. A scatter plot was used to visualize the trends of thiosulphate, methanol and its degradation product profiles throughout the experiment time.

## RESULTS

The main metabolites in the batch experiments were sulphate, hydrogen sulphide and carbon dioxide. The accumulation of sulphate, sulphide and carbon dioxide were related to the degradation of methanol and thiosulphate in the system. Their accumulation was also directly related with the growth of biomass in the system. The profiles of each metabolite, degradation of the two substrates and the biomass growth profile are presented below.


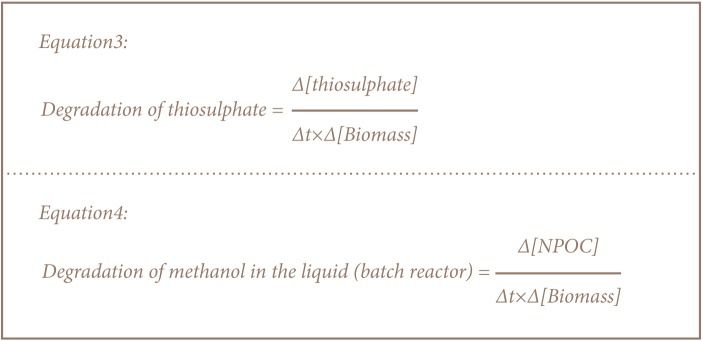


### Thiosulphate Degradation Profiles

**Figure 1:**
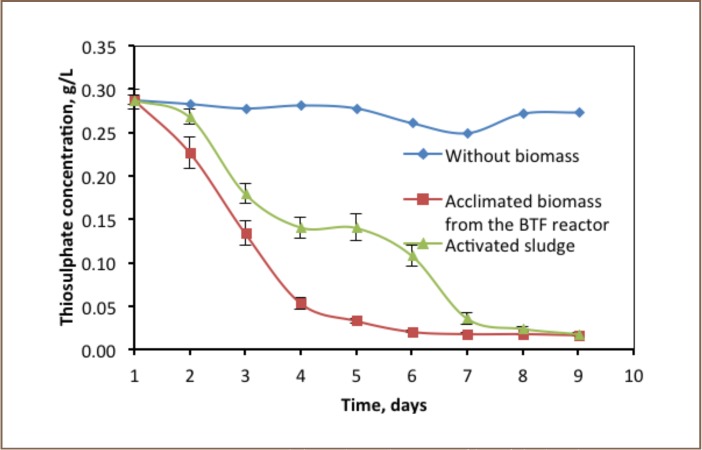
Thiosulphate degradation profiles of the batch reactor (n=2)

The degradation trends of thiosulphate in both batch reactors were directly correlated with the presence of biomass activity (*Figure 1*). Thiosulphate biodegradation in the batch reactor operated without biomass was not observed. However, the concentration was decreased from 0.29 to 0.27 g/L by speciation. As shown in Figure 1, there was a sharp decrease in concentration of thiosulphate from 0.29 to 0.05 g/L in the reactor operated with acclimated biomass taken from the biotrickling filter reactor. It was reduced gradually to 0.02 g/L. A total amount of 93.9% of thiosulphate used in the reactor was degraded by the microorganism within 9 days. In contrast to the experiment with acclimated biomass, the thiosulphate reduction using biomass of activated sludge was slower. Its concentration decreased gradually from 0.29 to 0.02 g/L. The reason for this is that the biomass from activated sludge took time to acclimatise itself to the new environment.

### Non-Purgeable Organic Carbon

**Figure 2:**
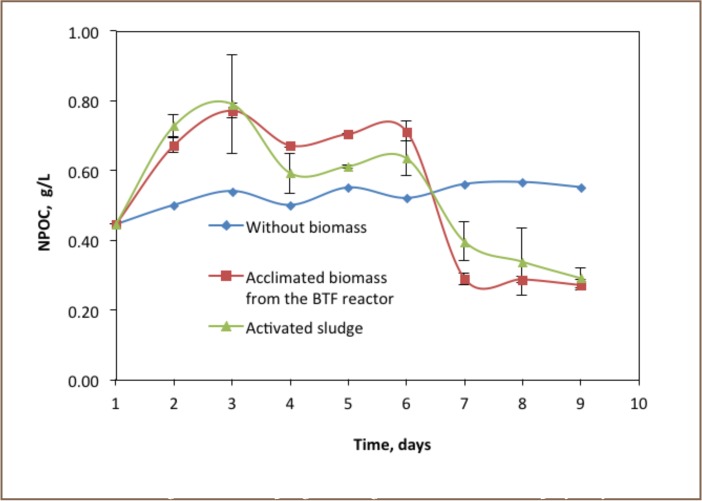
Non-purgeable organic carbon (NPOC) profile of the batch experiment (n=2)

All batch reactors contained 0.44 g/L of non-purgeable organic carbon before the experiment was started. As shown in Figure 2, the trends of NPOC degradation were not clearly understood for the first two days. The increasing NPOC trends were anticipated due to the growth of biomass community in the first three days in the two reactors. After 3 days, the NPOC value in the batch experiment operated with activated sludge was decreased sharply by 25% and NPOC decreased from 0.79 to 0.59 g/L. The NPOC value in the biotrickling filter acclimated biomass was also decreased from 0.79 to 0.67 g/L. The profile became constant for three days in both batch reactors containing biomass. However, the amount of NPOC was greater in the acclimated biomass, which was directly related to the amount of biomass in the reactor. NPOC in the batch reactor operated with acclimated biomass decreased to 0.29 g/L. A similar trend was observed for the other reactor operated with activated sludge as a biomass source. As a whole, it was observed that the highest degradation rate was recorded in the batch reactor containing biotrickling filter acclimated biomass. In addition, 64.8% of total NPOC was degraded with the action of acclimated biomass in 9 days. In addition, 63.2% of the NPOC had been degraded by activated sludge biomass. There was almost no change in degradation of methanol in the reactor without biomass. Thiosulphate was the limiting substrate when the methanol gas was not fully degraded.

### Carbon Dioxide Profile

**Figure 3:**
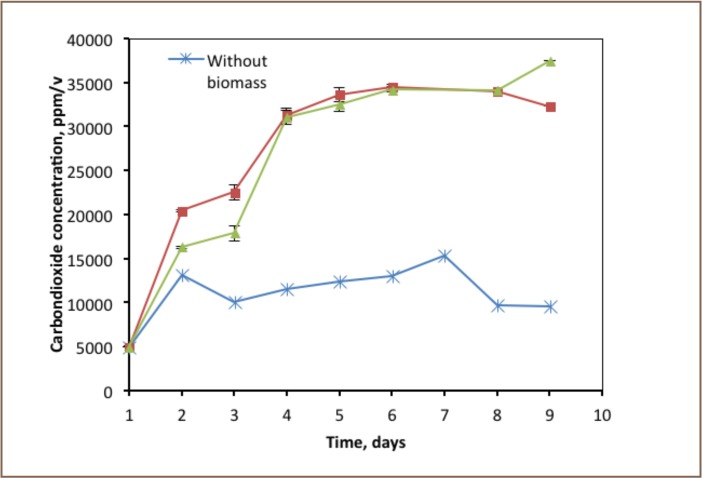
Carbon dioxide profile of the batch experiment (n=2)

The production of carbon dioxide shows a different trend. As illustrated in Figure 3, the initial concentration of carbon dioxide was about 4.9×10^3^ ppm/V. The batch operation without biomass showed no production of carbon dioxide. However, there was an increase in carbon dioxide gas in the experiment operated with acclimated biomass from the biotrickling filter reactor and biomass of activated sludge. The trends of carbon dioxide in the acclimated biomass showed higher production of carbon dioxide for the first 6 days as compared to the biomass of activated sludge. The maximum amount of 4.1×10^4^ ppm/V carbon dioxide was produced on day eight of the experiment. After that, it decreased sharply to 3.2×10^4^ ppm/V. In contrast to the acclimated biomass, the carbon dioxide production rate in the experiment operated with activated sludge biomass was slower for the first three experiment days. It increased slowly from 3.1×10^4^ ppm/V to 3.4×10^4^ ppm/V, then it increased sharply to 5.1×10^4^ ppm/V. Finally, it declined to 3.7×10^4^ ppm/V. The difference between the results of the duplicate experiments for the reactors was not significant.

### Sulphate

**Figure 4:**
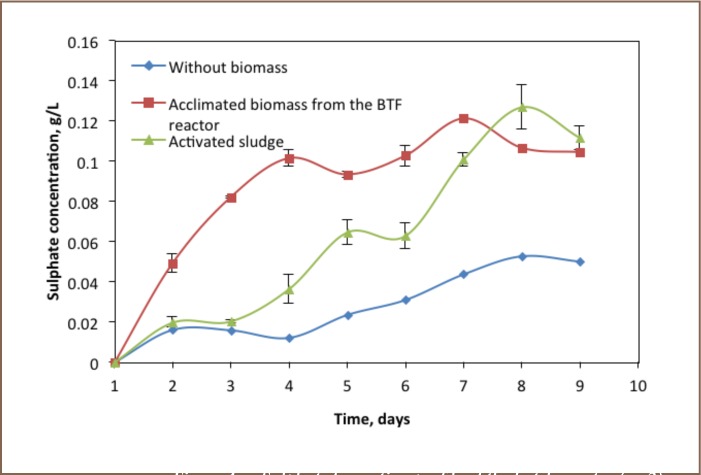
Sulphate formation profile of the batch reactor (n=2)

Sulphate was produced as one of the metabolites of thiosulphate degradation in the reactor. Its production rate was different in all three conditions. As shown in Figure 4, the rate of sulphate production in the batch reactors was related to the biomass activity. A batch reactor without biomass produces very little sulphate. The maximum production of 0.05 g/L was reached within 9 days. The production of sulphate in the reactor containing an acclimated biomass taken from the biotrickling filter reactor was increased sharply to 0.12 g/L after five days. There was slow growth of sulphate production in the batch experiment operated with activated sludge as a biomass source. It increased slowly until reaching a maximum sulphate amount of 0.13 g/L.

### Hydrogen Sulphide

**Figure 5:**
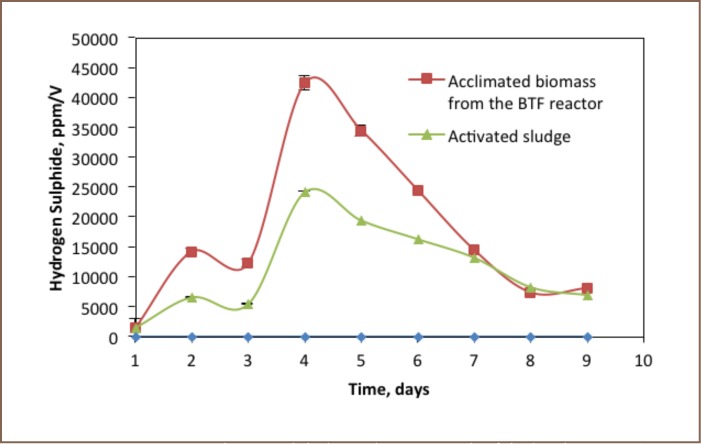
Hydrogen sulphide production trends of the batch reactor (n=2).

As shown in Figure 5, sulphide production appears correlated with biomass activity and thiosulphate degradation in the reactor. There was no hydrogen sulphide production observed in the reactor without biomass. Hydrogen sulphide gas production in the reactor using acclimated biomass increased sharply in the first four days and reached a maximum peak concentration of 4.2×10^4^ ppm/V, then declined slightly to 7.3×10^3^ ppm/V within the next five days. The production trend of hydrogen sulphide inside the batch reactor operated with activated sludge as a biomass source was similar to the batch reactor operated with acclimated biomass from the biotrickling filter reactor. However, the quantity of sulphide gas production using activated sludge was smaller. The maximum amount of hydrogen sulphide produced in the batch reactor containing activated sludge as a biomass source was 2.4×10^4^ ppm/V.

### Biomass Growth Profile

**Figure 6:**
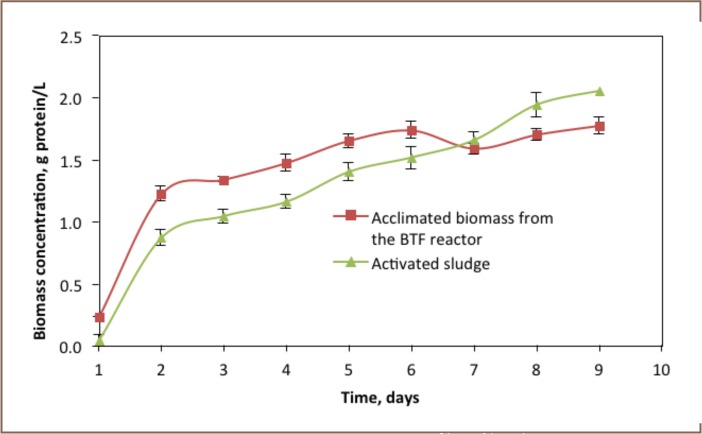
Biomass profiles of batch reactors

Initially, 0.04 and 0.23 g/L of biomass were present in the batch experiment operated using activated sludge and biotrickling filter reactor acclimated biomass, respectively. As shown in Figure 6, the biomass in both reactors increased exponentially for the first two days, then showed a gradual increase and finally levelled off. The biomass growth trend in the reactor operated by biotrickling filter acclimated biomass declined after 7 days. The maximum biomass amount recorded in this reactor was 1.7 g/L. However, the trend of biomass growth in the reactor containing activated sludge gradually increased until day 9. A maximum biomass amount of 2.1 g/L was recorded after 9 days in the reactor containing activated sludge.

### Biomass Growth Kinetics

The growth of biomass in the reactor correlated with the biodegradation profiles of thiosulphate and methanol. Methanol and thiosulphate biodegradation, biomass growth rate and yield coefficient expressed the kinetic activities of the biomass. The kinetic model is not detailed in the present article, but it was correlated with the rate of nutrient degradation with time. A monadic type of kinetic activity model was seen, where the growth of biomass was limited by the availability of food.

### Degradation of Thiosulphate

As shown in Figure 1, the biodegradation rate of thiosulphate in the batch reactor during 9 days of observation ranged from 0.02 to 0.80 per day. However, the activities of the two biomasses differed. The maximum thiosulphate degradation rate of 0.8 g thiosulphate per gram of biomass was achieved in the batch experiment operated with a biomass taken from a biotrickling filter.

### Degradation of Methanol

The biodegradation rate of methanol in the batch experiment operated with a biomass from previously used biotrickling filter was 0.02 to 0.74 mg/L methanol per mg/L biomass per day. However, more methanol was degraded in the reactor operated with the activated sludge as a biomass source. The degradation rate of NPOC in the reactor ranged from 0.52 to 1.50 mg/L methanol per mg/L biomass per day.

### Biomass Growth Trends

The growth rate of biomass in the batch experiment was analysed in terms of biomass accumulation in the reactor. The biomass growth rate (μ) observed in the batch reactor ranged from 0.04 to 1.7g biomass per day. The growth rate in the first days of the experiment was very fast, about 1.7 g biomass per day. At the last day of the experiment, the biomass growth rate was 0.04g per day. The observed biomass yield coefficient in the reactor was about 0.7 (g biomass)·(g methanol)^−1^.

## Discussion

Methanol is a volatile organic compound which is produced naturally, as well as from anthropogenic activities such as the pulp and paper industrial process. Its acute occupational exposure limits for workers and nearby residents are very small and the release of methanol from industrial activities needs to be properly managed. A small dose leads to blindness and its effects persist in the human body for a long time. Industries need to have appropriate and cost effective treatment technologies that do not create another intermediate hazardous product. Biological treatment technology is a lower cost treatment method that can be implemented in developing countries. However, there has been a shortage of information on biokinetic activity in terms of biodegradation rate and biomass yield constant. In addition, there is a shortage of cheap substrate to act as an electron acceptor. In the pulp and paper industry, thiosulphate is an intermediate sulphur compound found as a by-product of paper and pulp processing formed from the oxidation of sulphides and during the reduction of sulphate to sulphides and/or elemental sulphur. In the industry, thiosulphate is produced from the oxidation of sodium sulphide containing black liquor or released from the cooking liquor of Kraft's process. As it was found out in this experiment, it can serve as a good electron acceptor for methanol oxidation. Methanol is a suitable carbon source for bacteria during the reduction of thiosulphate and sulphate using sulphur reducing bacteria. Methanol was converted to carbon dioxide and water, and thiosulphate was converted into sulphate and sulphides.

### Degradation of Methanol

The degradation of methanol in the batch reactors was correlated with the production of carbon dioxide and availability of thiosulphate and sulphate, an electron acceptor. Degradation of methanol was attributed to the production of carbon dioxide and growth of biomass. As also explained in Goorissen, Stams and Hansen in a sulphidogenic process of anaerobic metabolism, methanol could be used as an electron donor for sulphur reducing bacteria or degraded by the combined activity of other microorganisms and changed to carbon dioxide.^16^,^18^ The same author also stated that its conversion pathway to carbon dioxide in the presence of sulphate or thiosulphate is energetically more favourable than the production of acetate or methane. The results of the NPOC profile (Figure 2) indicated that increasing growth of biomass increased the NPOC value for the first three days. However, the trends of NPOC sharply decreased due to increasing biodegradation of methanol in the reactor. The result implied that methanol concentration in the reactor was reduced from 0.44 g/L to 0.27 g/L, and it was converted to carbon dioxide and biomass cells. Approximately 63.2% of the initial NPOC used in the batch reactor was biologically degraded.

### Biodegradation of Thiosulphate

In the sulphidogenic process in an anaerobic environment, thiosulphate was biologically degraded to sulphide and sulphate by the action of sulphur reducing bacteria. Then, sulphate was reduced to sulphide. Sulphate and sulphide were the main metabolites of the thiosulphate biodegradation process. It was also found that under anaerobic conditions, thiosulphate was either disproportionate to sulphate and sulphide or directly reduced to sulphide by the action of sulphur reducing bacteria.^5^,^14^ In this experiment, the maximum amount of 0.80 mg/L of thiosulphate was biologically degraded per mg/L of biomass per day.

### Biomass Growth Kinetics in the Batch Experiment

The growth of biomass in the batch experiment correlated with the accumulation of sulphate and sulphide in the reactor. The production of sulphide and sulphate in the first few days of the experiment was very fast due to exponential growth of the biomass. The production of hydrogen sulphide declined at the sixth day due to depletion of thiosulphate substrate. The increasing nutrient competition between biomass in the system at this stationary phase reduced the biomass growth rate. As also found in Jorgensen and Bak F, the activity of sulphate reducers on thiosulphate degradation was very fast and sulphide and sulphate species were formed.^17^Compared to the activity of biotrickling filter acclimated biomass, biokinetic activity of activated sludge in the lag phase of the batch experiment was low, indicating that it took some time to acclimatise to the new methanol environment. However, there was no lag time observed for the biomass that was taken from the previously used biotrickling filter reactor. The bacteria automatically started their reduction work when they were mixed with the substrates containing nutrients. The biomass growth rate (μ) observed in the batch experiment was between 0.04 to 1.7 per day, and showed a similar growth trend of mixed culture bacteria studied by Jorgensen and Bak F who observed biomass growth rates between 1.2 to 3.84 per day with isolated bacteria from a mixed culture.^17^

## Conclusions

The simultaneous biodegradation of methanol and thiosulphate showed promising results for the treatment of both methanol and thiosulphate from waste stream under ambient conditions. The finding of methanol degradation in the presence of thiosulphate suggests that it is possible to clean toxic gas from the point of generation at large scale in the paper and pulp industries. This will ensure the ambient concentration level of toxic methanol gas surrounding to those industries where methanol is generated. The biomass used methanol as a carbon source and thiosulphate as an electron acceptor at a temperature of 20°C, pH 8.0 and pressure of 1 atm. Biodegradation of thiosulphate is very fast and is a limiting substrate for the biodegradation of methanol. Methanol can potentially be biologically treated in the presence of thiosulphate at ambient conditions in slightly alkaline conditions.
